# TRANSITIONAL CARE OUTCOMES IN VETERANS RECEIVING POST-ACUTE CARE IN A SKILLED NURSING FACILITY

**DOI:** 10.1093/geroni/igz038.2683

**Published:** 2019-11-08

**Authors:** Robert Burke, Anne Canamucio, Thomas Glorioso, Anna Baron, Kira Ryskina

**Affiliations:** 1 Center for Health Equity Research and Promotion, Corporal Crescenz VA Medical Center, Philadelphia, Pennsylvania, United States; 2 Center of Innovation for Veteran-Centered and Value-Driven Care Rocky Mountain Regional VA Medical Center, Aurora, Colorado, United States; 3 Colorado School of Public Health, University of Colorado Anschutz Medical Campus, Aurora, Colorado, United States; 4 Division of General Internal Medicine, Department of Medicine, University of Pennsylvania Perelman School of Medicine, Philadelphia, Pennsylvania, United States

## Abstract

More than 200,000 Veterans transition between hospital and skilled nursing facility (SNF) annually. Capturing outcomes of these transitions has been challenging because older adult Veterans receive care at VA and non-VA hospitals, and four different kinds of SNFs: VA-owned and -operated Community Living Centers (CLCs), VA-contracted community nursing homes (CNHs), State Veterans Homes (SVHs), and non-VA community SNFs. We used a novel data source which concatenates VA, Medicare, and Medicaid data into longitudinal episodes of care for Veterans, to calculate the rate of adverse outcomes associated with the transition from hospital to SNF in all enrolled Veterans age 65 and older undergoing this transition 2012-2014. The composite primary outcome included Emergency Department (ED) visits, rehospitalizations, and mortality (not in the context of hospice) within 7 days of hospital discharge to SNF. We used multivariable logistic regression to adjust for Veteran and hospital characteristics and hospital random effects. In the 388,339 Veterans discharged from 1502 hospitals in our sample, we found more than 4 in 5 Veteran transitions (81.7%) occurred entirely outside the VA system. The overall 7-day outcome rate was 10.7%. After adjustment, VA hospitals had lower adverse outcome rates than non-VA hospitals (OR 0.80, 95% CI 0.74-0.86). VA hospital-CLC transitions had the lowest adverse outcome rates; in comparison, non-VA hospital-CNH (OR 2.51, 95% CI 2.09-3.02) and non-VA hospital-CLC (OR 2.25, 95% CI 1.81-2.79) had the highest rates. These findings raise important questions about the VA’s role as a major provider and payer of post-acute care in SNF.

